# Single-dose mucosal replicon-particle vaccine protects against lethal Nipah virus infection up to 3 days after vaccination

**DOI:** 10.1126/sciadv.adh4057

**Published:** 2023-08-04

**Authors:** Stephen R. Welch, Jessica R. Spengler, Sarah C. Genzer, JoAnn D. Coleman-McCray, Jessica R. Harmon, Teresa E. Sorvillo, Florine E. M. Scholte, Sergio E. Rodriguez, T. Justin O’Neal, Jana M. Ritter, Georgia Ficarra, Katherine A. Davies, Markus H. Kainulainen, Elif Karaaslan, Éric Bergeron, Cynthia S. Goldsmith, Michael K. Lo, Stuart T. Nichol, Joel M. Montgomery, Christina F. Spiropoulou

**Affiliations:** ^1^ Viral Special Pathogens Branch, Division of High Consequence Pathogens and Pathology, Centers for Disease Control and Prevention, Atlanta, GA 30329, USA.; ^2^ Infectious Disease Pathology Branch, Division of High Consequence Pathogens and Pathology, Centers for Disease Control and Prevention, Atlanta, GA 30329, USA.

## Abstract

Nipah virus (NiV) causes a highly lethal disease in humans who present with acute respiratory or neurological signs. No vaccines against NiV have been approved to date. Here, we report on the clinical impact of a novel NiV-derived nonspreading replicon particle lacking the fusion (F) protein gene (NiVΔF) as a vaccine in three small animal models of disease. A broad antibody response was detected that included immunoglobulin G (IgG) and IgA subtypes with demonstrable Fc-mediated effector function targeting multiple viral antigens. Single-dose intranasal vaccination up to 3 days before challenge prevented clinical signs and reduced virus levels in hamsters and immunocompromised mice; decreases were seen in tissues and mucosal secretions, critically decreasing potential for virus transmission. This virus replicon particle system provides a vital tool to the field and demonstrates utility as a highly efficacious and safe vaccine candidate that can be administered parenterally or mucosally to protect against lethal Nipah disease.

## Teaser

Rapid protection against lethal Nipah virus infection after mucosal replicon particle vaccination in hamsters and mice.

## INTRODUCTION

Nipah virus (NiV), a bat-borne zoonotic henipavirus, causes severe and lethal respiratory and neurological disease in humans with case fatality rates up to 80% (*1*). A large outbreak of encephalitis in pig farmers in Malaysia in 1998–1999 led to the discovery of NiV and the identification of the NiV-Malaysia strain (*2*). Since 2001, nearly annual outbreaks of NiV infection have occurred in Bangladesh and India, caused by the related strain NiV-Bangladesh (*3*, *4*). Bats of the *Pteropus* genus (flying foxes) are the main natural reservoir of NiV and are found throughout some of the most densely populated regions of the world (*5*, *6*).

NiV can transmit to humans via direct contact with infected animals or their body fluids (blood, urine, or saliva) (*7*); exposure may also occur via consumption of food products, such as palm sap or fruit, contaminated with body fluids of infected animals (*8*). Symptoms in humans typically appear 4 to 14 days after virus exposure, initially presenting as nonspecific signs like fever and headache that can rapidly progress to severe or lethal respiratory disease. NiV infection can also result in acute, late-onset, or relapsed neurological disease, including in individuals that had acute nonencephalitic or asymptomatic infection (*9*). Furthermore, a spectrum of neuropsychiatric sequelae of NiV-induced encephalitis is described (*10*), including depression, personality changes, and chronic fatigue syndrome, indicating notable morbidity associated with nonfatal disease.

The initial outbreak in Malaysia and Singapore was linked to disease in pigs (*11*, *12*) and required the culling of almost a million pigs in efforts to control the outbreak. Subsequent experimental studies have confirmed pigs’ susceptibility to infection and characterized the corresponding spectrum of disease in these animals (*13*–*15*). While clinical disease is limited in most species, henipaviruses have notably broad species tropism; numerous animals have been identified as susceptible to infection in nature and in experimental studies [reviewed in (*16*–*20*)]. In addition, there are several well-characterized laboratory animal models of henipavirus disease. Frequently used models of NiV disease are hamsters, ferrets, and African green monkeys; all three models develop systemic vascular disease and parenchymal infection in a wide variety of organs and recapitulate both the respiratory and neurological disease observed in humans (*20*).

No therapeutics or vaccines are currently licensed for NiV infection. Given the severity of disease and the public health threat posed by NiV, substantial efforts are focused on developing safe and efficacious vaccine candidates for reactive use in outbreak response and for protecting high-risk communities in endemic regions (*21*, *22*). Vaccines evaluated to date predominantly use recombinant viral vectors and adjuvanted protein subunit vaccines targeting the fusion (F) and/or glycoprotein (G). Most candidates remain in preclinical development, although a single recombinant protein platform now completed phase 1 trials (*23*). We have previously reported the efficacy of virus replicon particle (VRP) vaccine platforms against viral hemorrhagic fevers when administered subcutaneously (*24*–*29*). Development of a VRP system has not been reported to date for henipaviruses nor has mucosal delivery of a VRP vaccine or of a NiV vaccine candidate. Here, to both provide a key molecular tool and to aid in crucial NiV vaccine development efforts, we developed and characterized a VRP lacking the F protein gene and evaluated its use as a novel mucosal vaccine platform against NiV infection and disease.

## RESULTS

### Generation and characterization of a nonspreading NiV VRP lacking the F gene

**Fig. 1.**
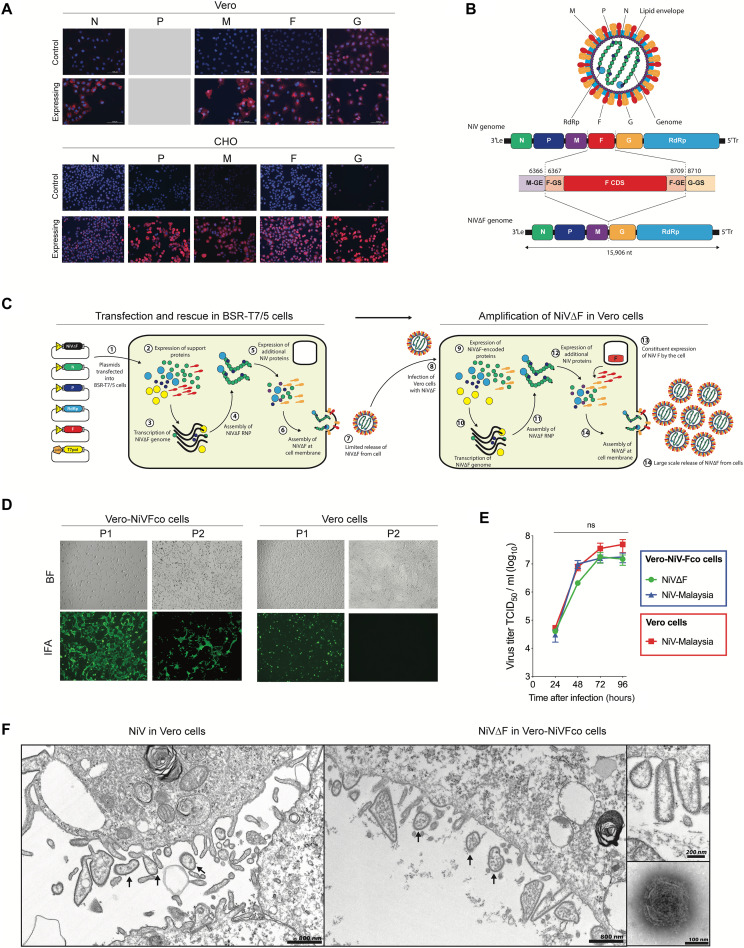
A NiV virus replicon particle can be efficiently grown in a cell line supplying F protein in trans. (**A**) Constitutive expression of NiV nucleoprotein (N), phosphoprotein (P), matrix protein (M), fusion protein (F), and glycoprotein (G) in Vero and CHO cells, confirmed by immunofluorescent staining showing viral proteins (red). Cells were also stained with 4′,6-diamidino-2-phenylindole (blue) to confirm viability. (**B**) Schematic showing design of NiV∆F genome. GE, gene end; GS, gene start; RdRp, RNA-dependant-RNA-polymerase; CDS, coding sequence; Le, leader sequence; Tr, trailer sequence. (**C**) Schematic showing the generation of NiV∆F. All required plasmids were transfected into BSR-T7/5 cells, and 2 days after transfection, cells were overlayed with the Vero-Fco cell line constitutively expressing codon-optimized NiV F protein. Five days after transfection, cell culture supernatants were harvested and clarified by centrifugation to collect NiV∆F VRP. (**D**) Both Vero and Vero-Fco cells were infected at multiplicity of infection (MOI) 1 with NiV∆F; 3 days postinfection (dpi), viral growth was confirmed by immunofluorescent staining (green). Clarified supernatants were passed onto fresh cells (P2), and 3 dpi, viral growth was again confirmed by immunofluorescent staining (green). IFA, immunofluorescence assay; BF, bright field. (**E**) Vero cells were infected with NiV (red squares), while Vero-Fco cells were infected with either NiV (blue triangles) or NiV∆F (green circles) (all MOI, 0.1). Viral titers were determined by TCID_50_ calculation 24 to 96 hours after infection. (**F**) Vero and Vero-Fco cells were infected with NiV and NiV∆F, respectively, at MOI of 0.01. After 4 days, the cells were processed for transmission electron microscopy to determine virion morphology. Particles of equivalent size and morphology were seen budding from both samples (arrows). No such particles were seen in mock-inoculated control samples. Inset shows a negatively stained electron microscopy image of a NiV∆F particle. ns, not significant.

NiV has a genome encoding six structural proteins [nucleoprotein (N), phosphoprotein (P), matrix protein (M), fusion protein (F), glycoprotein (G), and polymerase (L)], and three nonstructural proteins expressed within the P gene via RNA editing (V and W proteins) or an alternative open reading frame (C protein). To generate the VRP, we first engineered Vero-E6 and Chinese hamster ovary (CHO) cell lines to constitutively express one of the five proteins required for successful completion of the viral lifecycle (N, P, M, F, or G; Fig. 1A). Cell viability and protein expression levels were monitored for all protein-cell combinations, and expression was successful for all except P in Vero-E6 cells, which reduced cell viability. Next, we excised the entire F gene (gene start–coding sequence–gene end) from the existing reverse genetics plasmid that expresses the NiV genome (*30*), resulting in a plasmid capable of generating the truncated viral genome, termed NiV∆F (Fig. 1B). Modification of the NiV rescue system, in which the NiV∆F genome plasmid was used in place of authentic full-length NiV genome, and a further amplification step performed in Vero-E6 cells constitutively expressing the NiV codon-optimized F protein (Vero-Fco cells) resulted in successful generation of the NiV∆F VRP (Fig. 1C). A similar approach was initially attempted by removing the N gene, but we were unable to generate a NiV∆N VRP. Since generation of NiV∆F was successful on initial attempt and it appeared to be the most viable construct system, we focused on this platform for additional characterization and efficacy studies.

Next-generation sequencing (NGS) analysis of NiV∆F confirmed complete absence of the F gene in the viral genome. NiV∆F growth in Vero-Fco cells resulted in formation of syncytia characteristic of wild-type NiV, with similar cytopathic effects observed after a subsequent passage in Vero-Fco cells, indicating a viable viral lifecycle (Fig. 1D). Conversely, NiV∆F growth in standard Vero cells was constrained to the initial entry cell, with no viral growth observed in subsequent passages onto Vero cells. Growth kinetics showed that NiV∆F grown in Vero-Fco cells grew to similar titers as wild-type NiV in Vero cells (Fig. 1E). To confirm correct processing of the NiV Fco supplied in trans, we compared virion morphology of NiV and NiV∆F grown in Vero or Vero-Fco cells, respectively. NiV∆F VRPs budding from the cell membrane of Vero-Fco cells had the classic appearance of paramyxovirus virions and closely resembled authentic NiV virions budding from Vero cells (Fig. 1F). These data together confirm that NiV∆F VRP has the expected single-cycle nonspreading phenotype and can grow to titers similar to those of the parental wild-type virus when F is supplied in trans. In addition, the constitutively expressed F protein is processed correctly, allowing authentic viral budding and release processes to occur at the cell membrane.

### High safety margin and lack of inflammatory response to NiV∆F delivery in two highly sensitive models of NiV disease

**Fig. 2.**
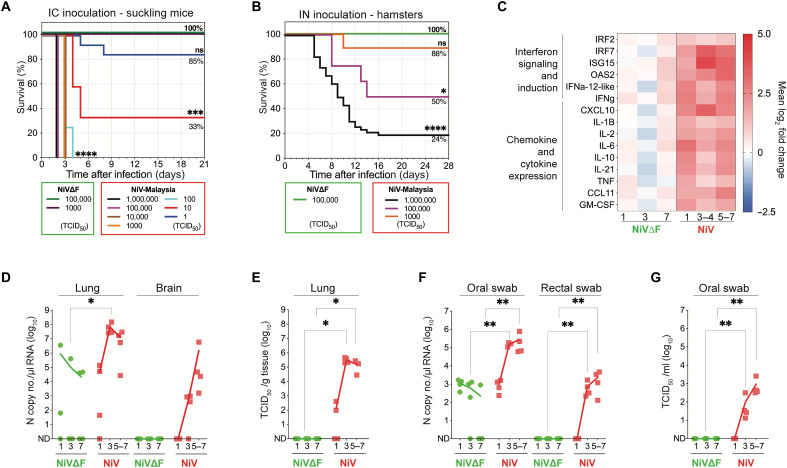
NiV∆F is nonspreading and noninfectious and confers a high safety margin in both suckling mice and hamsters. Kaplan-Meier survival curves of animals inoculated with NiV∆F: (**A**) Two- to 3-day-old suckling mice were intracerebrally (IC) inoculated with either 10^5^ or 10^3^ TCID_50_ of NiV∆F or one of seven dilutions of wild-type NiV ranging from 10^6^ to 10^0^ TCID_50_. (**B**) Five- to 7-week-old Syrian hamsters were IN inoculated with either 10^6^ TCID_50_ of NiV∆F or one of three dilutions of wild-type NiV ranging from 10^6^ to 10^3^ TCID_50_. Survival significance calculated by log-rank (Mantel-Cox test): ****P ≤ 0.0001; ***P ≤ 0.001; **P ≤ 0.01; *P ≤ 0.05. (**C**) Differential mRNA expression of select innate immune response genes in lungs of animals (**D** to **G**) was determined by RT-qPCR. (D) and (E) Lung and brain tissues and (F) and (G) mucosal swab samples (oral and rectal) were taken at euthanasia from hamsters serially euthanized 1, 3, or 7 days after inoculation with 10^6^ TCID_50_ of NiV∆F (green circles) or 1, 3, or 5 to 7 days after inoculation with wild-type NiV (red squares) to determine both vRNA levels (RT-qPCR) and quantify infectious virus titers (TCID_50_) in sample types in which NiV∆F RNA was detected. For samples analyzed in (C) to (G), a subset of wild-type NiV-infected animals did not survive to predetermined 7 dpi time point and were sampled earlier when end point criteria were reached (5 to 6 dpi). Significance was calculated by multiple *t* test: *P ≤ 0.05; **P ≤ 0.01.

Because using NiV∆F as a molecular tool or vaccine candidate outside of high-level biocontainment requires rigorous safety assessments, we conducted a series of studies to confirm the nonspreading, noninfectious phenotype of NiV∆F and its corresponding safety in multiple animal models of lethal NiV disease. We first compared NiV∆F infection to wild-type NiV infection in the highly sensitive suckling mouse model. Groups of 9 to 14 animals (2 to 3 days old) were inoculated intracerebrally (IC) and monitored daily for survival and clinical signs. All mice inoculated with 10^6^ to 10^2^ median tissue culture infectious dose (TCID_50_) of wild-type NiV succumbed by 4 days postinfection (dpi) (*P* ≤ 0.0001); mortality was reduced only with very low-dose inoculations: 66% at 10^1^ TCID_50_ (*P* = 0.0008) and 15% at 10^0^ TCID_50_ (Fig. 2A). Mice inoculated with 10^5^ TCID_50_ wild-type NiV uniformly succumbed in 2 days. However, when inoculated with 10^5^ TCID_50_ of NiV∆F, all mice survived with no clinical signs observed over the 21-day study period (*n* = 12).

We next used the well-characterized Syrian hamster NiV disease model. Intranasal (IN) inoculation with 10^6^ TCID_50_ of wild-type NiV (*n* = 46; 5- to 7-week-old hamsters; historical in-house data) results in 76% mortality (*P* ≤ 0.0001), with all animals exhibiting clinical signs at some point during the study (Fig. 2B). Again, however, hamsters (*n* = 20; 5 to 7 weeks old) inoculated IN with 10^6^ TCID_50_ of NiV∆F exhibited complete absence of clinical signs of disease throughout the 28-day study period, closely matching all clinical parameters of mock-challenged hamsters (fig. S1).

In addition, because inflammatory events crucial for triggering strong antigen-specific acquired immune responses may also lead to the development of vaccination side effects (*31*), we compared early inflammatory responses to NiV∆F to those induced by wild-type NiV. We assessed expression of 15 selected inflammatory and antiviral genes in lung tissue of IN-infected hamsters (Fig. 2C). After wild-type NiV infection, IRF7 (8.08-fold mean increase), ISG15 (14.87-fold mean increase), and CXCL10 (8.68-fold mean increase) were the most strongly up-regulated genes, with levels peaking 3 to 4 dpi and remaining high five to seven dpi. In contrast, in NiV∆F-inoculated hamsters, expression of these genes changed minimally at equivalent time points: IRF7 (1.17-fold mean increase), ISG15 (1.68-fold mean increase), and CXCL10 (1.09-fold mean increase). A similar pattern was observed in the other genes analyzed, with up-regulation seen between 3 and 7 dpi in NiV-infected hamsters, while in NiV∆F-inoculated animals, the response that could lead to adverse events was minimal and transient.

### Transiently detected NiV∆F RNA in tissue and mucosal specimens is not infectious or associated with histopathological changes

To investigate dissemination of NiV∆F in vivo, hamsters were inoculated IN with either NiV∆F, wild-type NiV, or Dulbecco’s modified Eagle’s medium (DMEM; negative control), and groups (*n* = 4 each) were serially euthanized 1, 3, and 7 dpi (or earlier if animals met euthanasia criteria due to NiV disease). Tissues were collected for viral RNA (vRNA) quantification, virus isolation, and histopathological evaluation; swab specimens (oral and rectal) were collected to characterize mucosal shedding in this model (fig. S2). NiV∆F vRNA was detected in lung but not brain, and NiV∆F virions were not isolatable from either tissue, whereas NiV was detected in both tissues and isolated from lung (Fig. 2, D and E). vRNA levels in oropharyngeal samples taken from NiV∆F-inoculated hamsters at 4 and 7 dpi were significantly (*P* = 0.029) lower than vRNA levels in NIV-inoculated hamsters, and, as expected, NiV∆F could not be isolated. In rectal swabs, no NiV∆F RNA was detected after vaccination compared (*P* = 0.029) to rising levels detected 3 and 7 dpi in NiV-infected hamsters (Fig. 2, F and G).

**Fig. 3.**
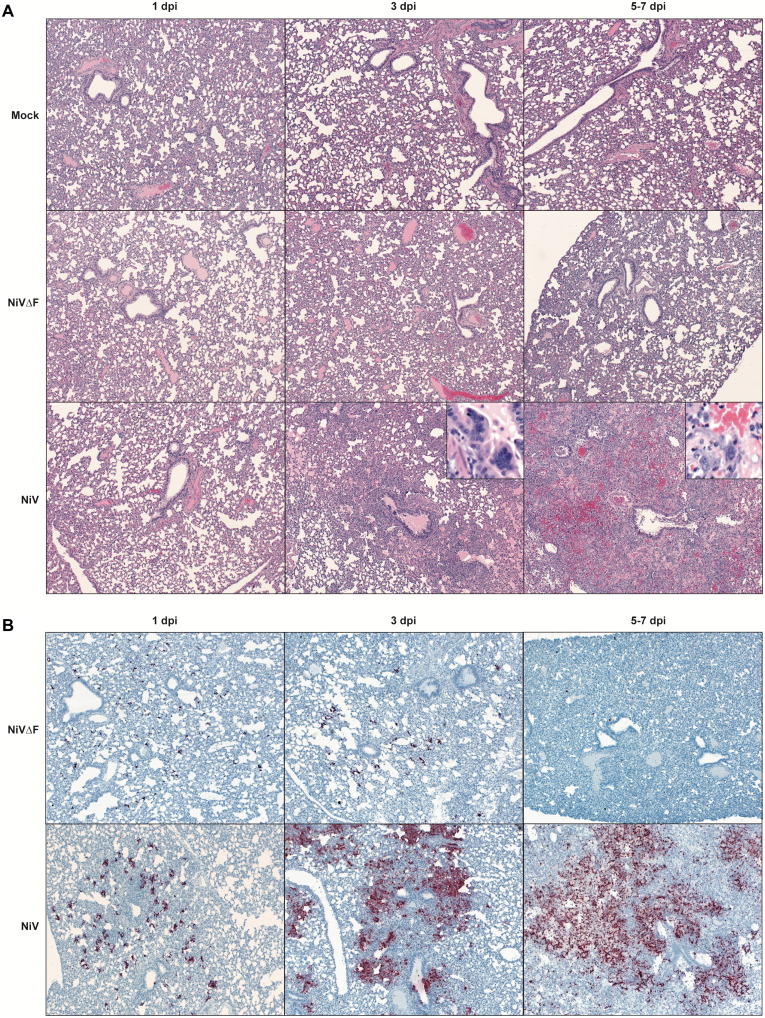
Absence of histopathology and nonspreading phenotype of NiV∆F in lung tissue from Syrian hamsters. Lung tissues from hamsters serially euthanized following inoculation with 10^6^ TCID_50_ of NiV∆F (1, 3, or 7 days), wild-type NiV [1, 3, or 5 to 7 days; subset of wild-type NiV-infected animals did not survive to predetermined 7 dpi time point and were sampled earlier when end point criteria were reached (5 to 6 dpi)], or DMEM only (mock-infected at 1, 3, or 7 days) were formalin fixed and evaluated via (**A**) hematoxylin and eosin staining to characterize tissue pathology and via (**B**) ISH to detect vRNA. Lungs of NiV∆F- and mock-inoculated animals showed no notable histopathologic changes at any time point, while lungs from NiV-inoculated animals displayed progressive inflammation, with epithelial syncytia (insets) compatible with paramyxoviral pneumonia. NiV∆F vRNA was detected by ISH 1 dpi and decreased by 7 days. In contrast, NiV vRNA was detected at relatively higher levels starting 1 day after inoculation and increased over time with severity of pneumonia.

Histopathological examination of tissues collected 1, 3, and 7 dpi indicated the absence of pathology in all tissues examined from NiV∆F-inoculated animals (liver, spleen, gonad, kidney, heart, lung, eye, and brain) at all time points. In contrast, NiV infection resulted in inflammation and progressive bronchointerstitial pneumonia with syncytial cells characteristic of paramyxoviral infection seen by 7 dpi in all but one animal (Fig. 3A). This animal did not develop pneumonia but had a focus of neuronal degeneration and meningeal inflammation in the olfactory bulb of the brain. In situ hybridization (ISH) was used to examine vRNA over time in lung and select other tissues. NiV∆F vRNA was detected in lung 1 dpi and decreased by 7 dpi. In contrast, NiV vRNA was detected at comparatively higher levels 1 dpi, and ISH demonstrated increased staining in the pneumonic lungs at later time points (Fig. 3B). One sample collected 7 dpi from an NiV-infected animal had focal ISH staining in extrapulmonary tissues without overt pathologic changes (brain, liver, spleen, kidney, adrenal gland, and renal lymph node), and the abovementioned animal with focal brain pathology had extensive vRNA in rostral brain tissue.

### Parenteral or mucosal delivery of NiV∆F elicits a robust antibody response

**Fig. 4.**
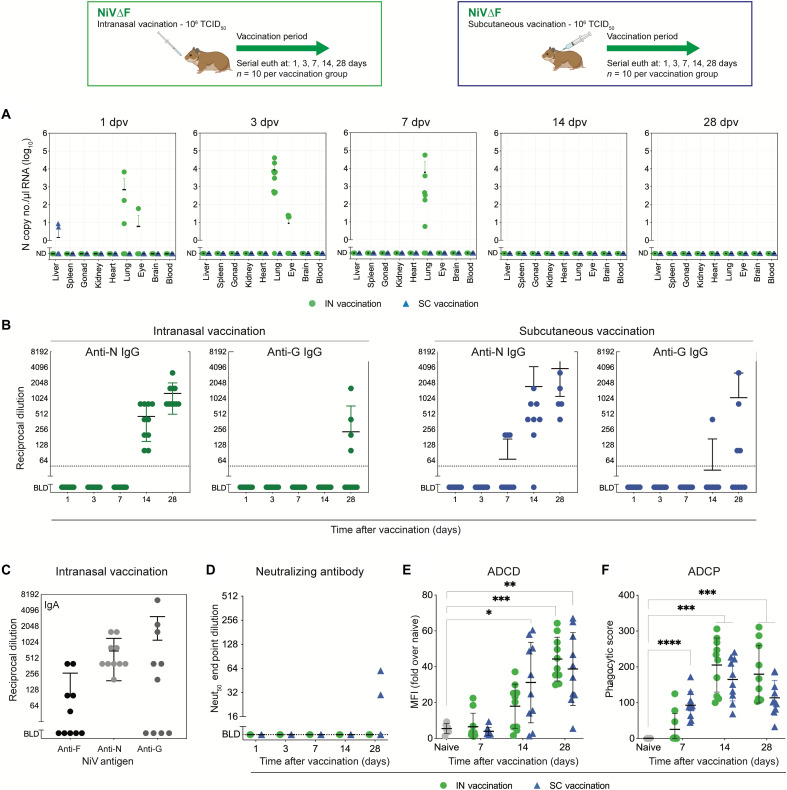
Humoral immunity after single-dose mucosal or subcutaneous delivery of NiV∆F. Five- to 7-week-old Syrian hamsters were inoculated either IN (green circles) or SC (blue triangles) with 10^6^ TCID_50_ of NiV∆F and euthanized 1, 3, 7, 14, or 28 days postvaccination (dpv; *n* = 10 per group). (**A**) NiV∆F vRNA tissue levels at each time point were determined by RT-qPCR. (**B**) IgG antibody titers against NiV N and G proteins were determined by enzyme-linked immunosorbent assay (ELISA) at all time points after vaccination. (**C**) For IN-vaccinated hamsters, IgA titers against NiV F, N, and G were determined by ELISA at 28 days postvaccination. (**D**) Neutralizing antibody titers against NiV strain Malaysia were evaluated 1, 3, 7, 14, and 28 days postvaccination. (**E**) Antibody-dependent complement deposition (ADCD) assay showing complement fixing activity of antibodies and (**F**) antibody-dependent cellular phagocytosis (ADCP) assay depicting phagocytic activity of antibodies were performed on plasma collected 7, 14, and 28 days postvaccination. In all panels, individual values are shown, with bars representing mean and SD. Significance calculated by multiple *t* test: *P ≤ 0.05; **P ≤ 0.01; ***P ≤ 0.001; ****P ≤ 0.0001. ND, not detected; BLD, below limit of detection; MFI, mean fluorescence intensity.

To investigate NiV∆F tissue dissemination kinetics and characterize immunogenicity following peripheral and mucosal delivery, groups of hamsters were vaccinated either IN or subcutaneously (SC) with 10^6^ TCID_50_ and serially euthanized 1, 3, 7, 14, and 28 dpi (*n* = 10 per route per time point). Independently of inoculation route, NiV∆F delivery resulted in limited and transient tissue dissemination (Fig. 4A). In IN-inoculated animals, NiV∆F vRNA was detected by reverse transcription quantitative polymerase chain reaction (RT-qPCR) in the lungs up to 1 week after inoculation. Low levels (<100 genome copies) of NiV∆F were detected in the eyes of a minority of animals 1 and 3 dpi. In SC-inoculated animals, aside from low levels (<10 genome copies) detected in liver of two animals 1 dpi, no NiV∆F vRNA was detected.

Although vRNA detection in tissues was limited, antibody responses to NiV∆F were noted beginning 7 and 14 dpi in SC- and IN-inoculated animals, respectively. A minority of SC-vaccinated animals developed anti-N immunoglobulin G (IgG) responses 7 dpi, and all but one animal had anti-N IgG responses 14 dpi regardless of vaccination route (Fig. 4B). Independently of vaccination route, the anti-G IgG response was less pronounced, with only 40 to 50% of animals developing a detectable response by 28 dpi. In all cases, the anti-N response was stronger than the anti-G response. We also examined plasma IgA response 28 dpi in IN-vaccinated animals. As with IgG, all animals (*n* = 10) developed an anti-N IgA response; 6 of 10 and 5 of 10 also had detectable anti-G and anti-F IgA, respectively (Fig. 4C). Both vaccination routes resulted in almost complete absence of detectable antibody neutralizing activity, with only two animals (both SC-vaccinated) developing low-level neutralizing antibody responses (Fig. 4D).

To further investigate the functions of NiV∆F-elicited antibodies, we performed Fc-effector function assays. In an antibody-dependent complement deposition (ADCD) assay examining Igs targeting NiV N, an increased response correlated to the length of the vaccination period, with significant levels of complement deposition seen 28 dpi in both IN-inoculated (*P* = 0.0003) and SC-inoculated (*P* = 0.0018) animals compared to mock-vaccinated animals (Fig. 4E). We also detected significant phagocytic activity of antibodies 7, 14, and 28 dpi with NiV∆F (Fig. 4F). These data demonstrate that both IN and SC vaccinations with NiV∆F elicit a broad adaptive immune response with multiple Ig classes targeting N, F, and G.

### A single intranasal dose of NiV∆F up to 3 days before challenge protects hamsters from death and all clinical signs of disease

**Fig. 5.**
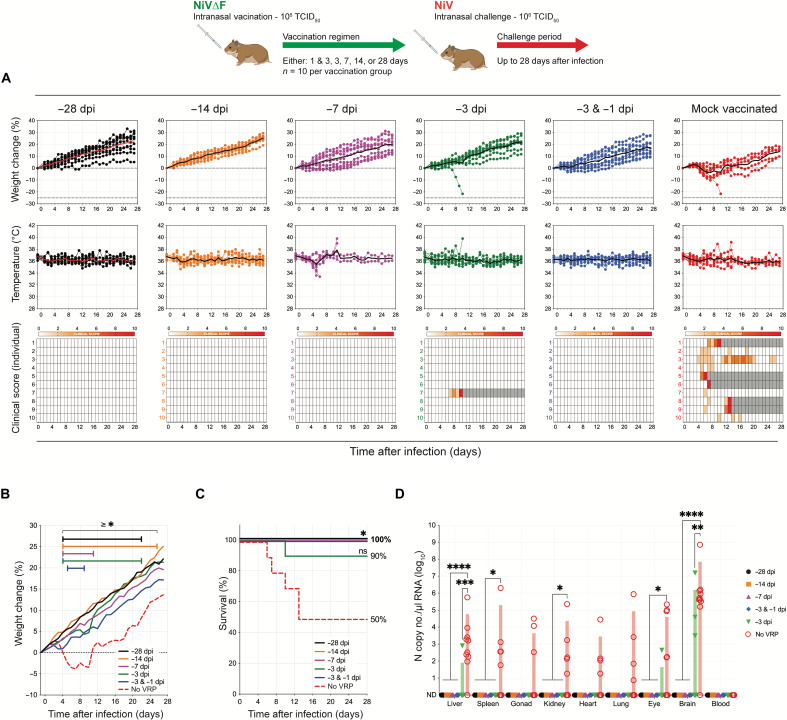
Mucosal NiV∆F vaccination protects Syrian hamsters from NiV clinical signs and lethality up to 3 days before IN challenge. Groups of 10 hamsters were vaccinated IN with 100 μl containing 10^6^ TCID_50_ of NiV∆F: once 28 (black), 14 (orange), 7 (purple), or 3 (green) days before challenge; or twice (blue) both 3 and 1 days before challenge. Mock-vaccinated animals (red) were given an equivalent IN volume of DMEM 28 days before challenge. All hamsters were challenged IN with 10^6^ TCID_50_ NiV strain Malaysia. (**A**) Percent weight change from baseline (taken −1 dpi); mean daily body temperatures; and individual daily clinical scores (from 0 to 10), with severity depicted by increased intensity of red. Animals scoring ≥ 10 were humanely euthanized; any animal that succumbed to disease before euthanasia was allocated a score of 10. Gray boxes indicate the end of monitoring/scoring due to euthanasia/death. Individual animals are represented as circles, with the solid line representing the daily mean. (**B**) Mean percent weight changes from baseline (taken −1 dpi; significance calculated by two-way analysis of variance (ANOVA): *P ≤ 0.05). (**C**) Kaplan-Meier curves showing survival of vaccinated and mock-vaccinated animals challenged with NiV strain Malaysia. Significance calculated by log-rank (Mantel-Cox test): *P ≤ 0.05; ns, not significant. (**D**) RT-qPCR detection of NiV vRNA in select tissues from hamsters vaccinated at indicated times and subsequently challenged. Individual animals from each group are represented. Error bars represent the means and SD. Significance calculated by *t* test: ****P ≤ 0.0001; ***P ≤ 0.001; **P ≤ 0.01; *P ≤ 0.05.

Protective efficacy was determined in hamsters vaccinated IN with NiV∆F at various intervals prechallenge: single dose (10^6^ TCID_50_) −3, −7, −14, or −28 dpi or two doses (10^6^ TCID_50_ at each time point) −1 and −3 dpi. Throughout the prechallenge vaccination period, no weight loss, fever, or clinical signs were observed in any group. Hamsters (9 to 11 weeks old) were challenged IN with 10^6^ TCID_50_ of NiV and monitored daily. All mock-vaccinated animals displayed significant weight loss along with clinical signs for one or more days of the 28-day challenge period, and 50% succumbed to infection (Fig. 5, A to C). In contrast, except for a single animal vaccinated once at 3 days before challenge that succumbed to disease 10 dpi, all hamsters vaccinated with NiV∆F survived; no significant weight loss, changes in body temperature, or any clinical signs were observed in vaccinees. vRNA levels were assessed at terminal end points or study completion. Overall, vaccination significantly reduced vRNA levels compared to mock vaccination. Animals vaccinated 28, 14, or 7 days before challenge, and those vaccinated with two doses 3 and 1 days before challenge, had no detectable NiV vRNA in any of the tested tissues at study completion (−28 dpi; Fig. 5D). The single animal that succumbed in the single-dose 3-day vaccine group had detectable, but significantly lower, NiV vRNA levels in the liver, eye, and brain only. Of the survivors in this group, two animals had detectable vRNA levels in the brain without displaying any clinical signs.

**Fig. 6.**
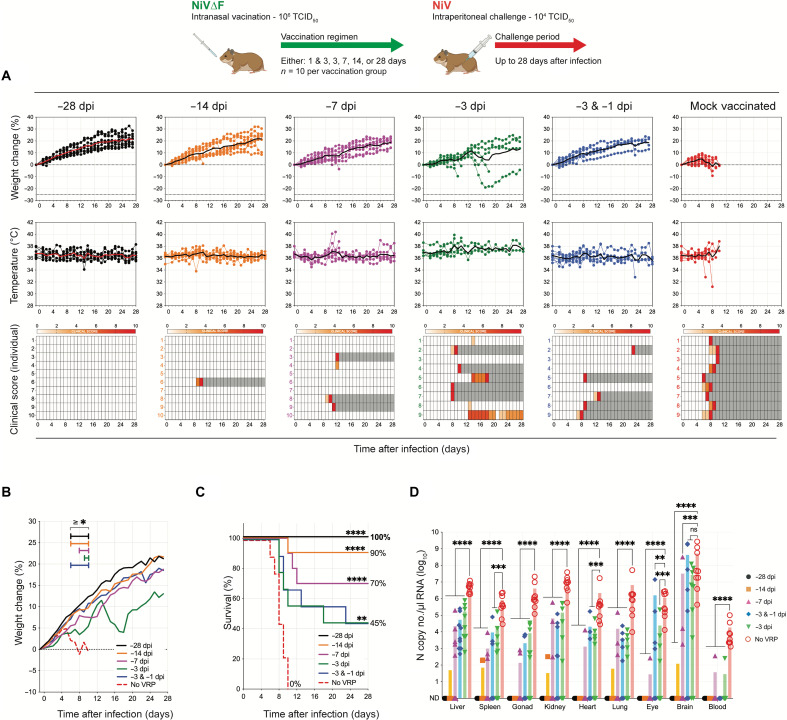
Mucosal NiV∆F vaccination confers high-level protection against disease and lethality in robust Syrian hamster intraperitoneal challenge model. Groups of 9 and 10 hamsters were vaccinated IN with 100 μl containing 10^6^ TCID_50_ of NiV∆F: once 28 (black), 14 (orange), 7 (purple), or 3 (green) days before challenge; or twice (blue) both 3 and 1 days before challenge. Mock-vaccinated animals (red) were given an equivalent IN volume of DMEM 28 days before challenge. All hamsters were challenged IP with 10^4^ TCID_50_ NiV strain Malaysia. (**A**) Percent weight change from baseline (taken −1 dpi); mean daily body temperature; and individual daily clinical scores (scored from 0 to 10), with severity depicted by increased intensity of red. Animals scoring ≥ 10 were humanely euthanized; any animal that succumbed to disease before euthanasia was allocated a score of 10. Gray boxes indicate the end of monitoring/scoring due to euthanasia or death. Individual animals are represented as circles, with the solid line representing the daily mean. (**B**) Mean percent weight changes from baseline (taken at −1 dpi; significance calculated by two-way ANOVA: *P ≤ 0.05). (**C**) Kaplan-Meier curves showing survival of vaccinated and mock-vaccinated animals challenged with NiV strain Malaysia. Significance calculated by log-rank (Mantel-Cox test): ****P ≤ 0.0001; **P ≤ 0.01. (**D**) RT-qPCR detection of NiV vRNA in select tissues from hamsters vaccinated at indicated times and subsequently challenged. Individual animals from each group are represented. Error bars represent the means and SD. Significance calculated by *t* test: ****P ≤ 0.0001; ***P ≤ 0.001; **P ≤ 0.01.

Although clinical signs were observed in all mock-vaccinated animals following IN challenge, mortality only reached 50%. Since older hamsters can be resistant to lethal henipavirus disease (*20*, *32*), we further evaluated the NiV∆F vaccine in the more stringent intraperitoneal (IP) challenge model (10^4^ TCID_50_) using the same vaccination periods and dosing regimens and daily monitoring (Fig. 6A). In this model, all mock-vaccinated hamsters displayed notable weight loss (Fig. 6B) and succumbed to disease (Fig. 6C), whereas improved clinical outcomes and significant increase in survival were seen in all vaccinated groups. Animals vaccinated 28 days before challenge displayed no clinical signs. As vaccine periods were shortened, some breakthrough lethal disease was observed: 1 of 10 (10%) and 3 of 10 (30%) of animals succumbed to disease after vaccination periods of 14 and 7 days, respectively. However, except for one hamster vaccinated 7 days before challenge that had only a single day with clinical signs, none of the surviving vaccinated animals in these groups developed any clinical signs throughout the study period. Four of the nine animals vaccinated with two doses (at −3 and −1 dpi) survived, and none displayed clinical signs throughout the challenge period. Four of nine hamsters vaccinated with a single dose 3 days before challenge survived: 1 displayed no weight loss or clinical signs, 2 had mild weight loss with 1 day of mild neurological clinical signs, and the remaining survivor displayed mild to moderate neurological signs for 12 days (13 to 25 dpi) but recovered by the end of study. Animals vaccinated 28 days before challenge again had no detectable vRNA in any of the tested tissues (Fig. 6D). With the shorter vaccination periods, vRNA was more widely detected, but levels were significantly lower than those in mock-vaccinated hamsters.

### NiV∆F vaccination reduces viral load in tissues and risk of transmission from mucosal secretions early in infection

To detail the effects of NiV∆F vaccination on infection and viral shedding and to assess comparative immune responses, we analyzed samples collected from vaccinated hamsters at early time points after challenge. Hamsters (*n* = 120) were vaccinated IN with NiV∆F (10^6^ TCID_50_) or mock-vaccinated with DMEM. To mirror the models used in efficacy studies, subsets were challenged with NiV either IN (10^6^ TCID_50_) or IP (10^4^ TCID_50_) 28, 14, 7, or 3 days after vaccination (all hamsters 9 to 11 weeks old at challenge). Groups (*n* = 4 per vaccination period per challenge route) were serially euthanized at one of the three predetermined end points (1, 4, or 6 dpi), and tissues were collected for virological and immunological analyses.

**Fig. 7.**
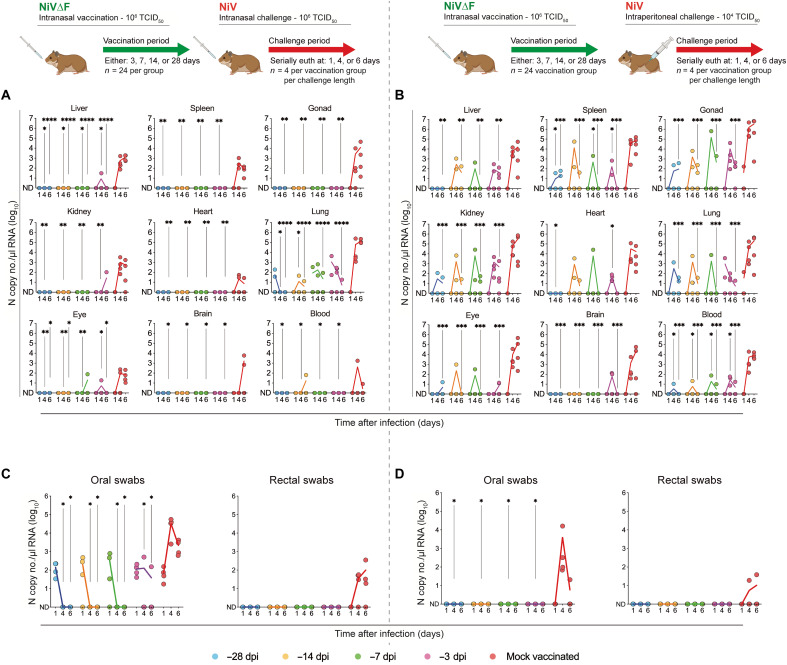
NiV∆F vaccination reduces NiV replication in tissues and mucosal membranes early in infection. Groups of hamsters were vaccinated IN once 28 (blue), 14 (orange), 7 (green), or 3 (purple) days before challenge or mock-vaccinated with DMEM (red) 28 days before challenge (total *n* = 120; *n* = 24 in each vaccination group; 12 males and 12 females; all age matched). Animals were challenged either IN with 10^6^ TCID_50_ (*n* = 60) or IP with 10^4^ TCID_50_ (*n* = 60) with NiV strain Malaysia. At 1, 4, or 6 dpi, select animals were euthanized for analysis (*n* = 40 per dpi; *n* = 4 per vaccination period per vaccination route per day; two males and two females per group). Represented are the NiV vRNA titers in select tissues (**A** and **B**) and in oral and rectal swabs (**C** and **D**) at each time point. In all panels, individual animals are represented as circles, with a solid line indicating the mean at each time point. In all panels, significance is indicated in relation to mock-vaccinated animals (red dots) from the same time point after challenge, calculated by log two-way ANOVA with Dunnett’s test to correct for multiple comparisons: ****P ≤ 0.0001; ***P ≤ 0.001; **P ≤ 0.01; *P ≤ 0.05.

Overall, viral loads in all tissues of vaccinated animals were significantly lower 4 and/or 6 dpi than in mock-vaccinated animals (Fig. 7, A and B). The magnitude of vRNA level reduction correlated with timing of NiV∆F vaccination; administration earlier before challenge conferred more uniform and pronounced reduction in vRNA levels. Notably, vRNA was largely undetectable in hamsters challenged IN regardless of vaccination period. While detection was more pronounced in IP-challenged animals, likely because of more direct distribution of the virus to visceral organs following parenteral exposure, it remained significantly reduced in vaccinated animals compared to mock-vaccinated ones. Vaccination also reduced vRNA levels in oral and rectal mucosa swab specimens (Fig. 7, C and D). In IP-challenged hamsters, no oral swabs from vaccinated hamsters contained detectable vRNA (Fig. 7D). In IN-challenged hamsters, vRNA was detected in oral swabs 1 dpi. However, except for one of four animals vaccinated 3 days before challenge, no IN-challenged hamsters vaccinated ≥3 days before challenge had detectable vRNA 4 or 6 dpi (Fig. 7C). In both IN- and IP-challenged hamsters, vRNA was detected in rectal swabs of unvaccinated controls 4 and 6 dpi; no rectal vRNA was detected in NiV∆F-vaccinated hamsters (Fig. 7, C and D).

### Protection by NiV∆F vaccination is associated with increased Fc-dependent antibody effector functions early after challenge

Rapid induction of ISGs was previously demonstrated to contribute to post-exposure protection by an Ebola virus–like particle vaccine (*33*). Like Ebola virus, NiV is known to impair type I interferon (IFN) signaling and delay induction of antiviral ISGs (*34*–*38*). Given the rapidity of protection we saw after short vaccination periods (i.e., 3 days), we postulated that differential innate responses in the context of infection may also contribute to short-course VRP-mediated protection against NiV. To assess the contribution of nonspecific innate immune responses to protection, plasma collected from hamsters serially euthanized 1, 4, or 6 dpi (either IN or IP) was analyzed by RT-qPCR to quantify inflammatory and antiviral gene expression. Earlier, we found that immune responses in NiV∆F-vaccinated animals were minimal and transient (Fig. 2C). Here, following virus challenge, few differences in innate immune responses were seen between vaccinated and unvaccinated hamsters (fig. S3), indicating that a divergence in innate immune responses early in infection cannot be associated with differential outcomes.

**Fig. 8.**
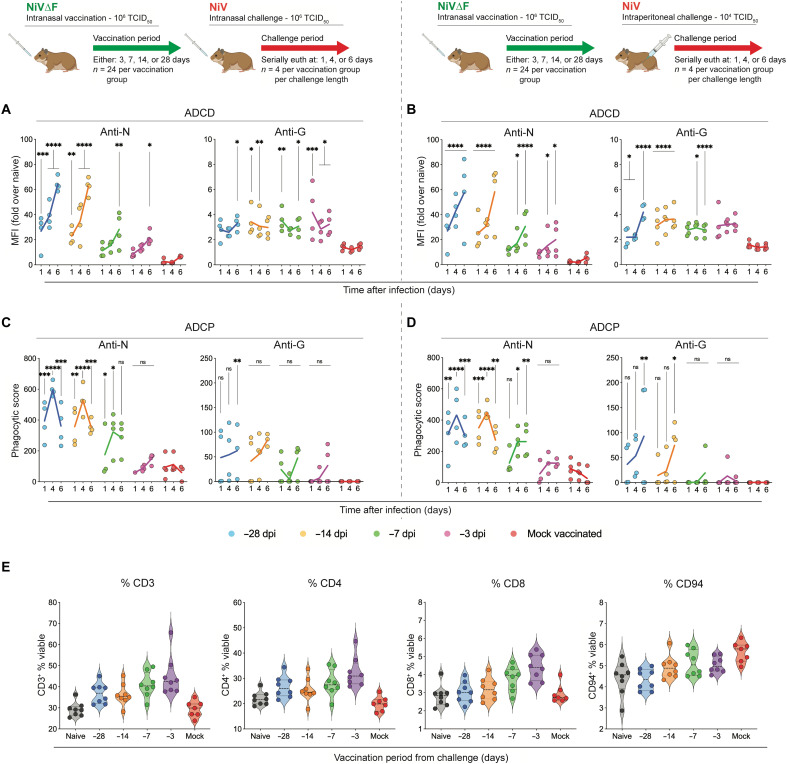
NiV∆F vaccination enhances Fc-mediated antibody effector functions. Groups of hamsters were vaccinated IN once 28 (blue), 14 (orange), 7 (green), or 3 (purple) days before challenge, or mock-vaccinated with DMEM (red) 28 days before challenge (total, *n* = 120; for each vaccination group, *n* = 24; 12 males and 12 females; all age matched). Animals were challenged either IN with 10^6^ TCID_50_ (*n* = 60) or IP with 10^4^ TCID_50_ (*n* = 60) with NiV strain Malaysia. At 1, 4, and 6 dpi, select animals were euthanized for analysis (*n* = 40 per dpi; *n* = 4 per vaccination period per vaccination route per day; two males and two females per group). (**A** and **B**) ADCD and (**C** and **D**) ADCP against N and G antigens. In all panels, individual animals are represented as circles, with a solid line indicating the mean at each time point. In all panels, significance is indicated in relation to mock-vaccinated animals (red dots) from the same time point after challenge, calculated by log two-way ANOVA with Dunnett’s test to correct for multiple comparisons: ****P ≤ 0.0001; ****P ≤ 0.001; **P ≤ 0.01; *P ≤ 0.05; ns, non significant. MFI, mean fluorescence intensity. (**E**) Single-cell suspensions of splenocytes collected 6 days after NiV challenge (IP or IN) from hamsters either mock-vaccinated (DMEM only) or VRP-vaccinated IN for indicated vaccine intervals before challenge were stained for fluorescence-activated cell sorting analysis. T cells were identified and stained for expression of CD3, CD4, and CD8, with levels depicted as % of viable cells. NK cells were identified and stained for expression of CD94 (gating strategy shown in fig. S4). Samples from NiV-infected hamsters were compared to those obtained from naïve (unvaccinated and uninfected) hamsters.

Fc-dependent antibody effector functions have been shown to contribute to antibody-mediated protection against disease with other viruses, including HIV (*39*), influenza (*40*), severe acute respiratory syndrome coronavirus 2 (SARS-CoV-2) (*41*), and Ebola virus (*42*). Earlier, we described that NiV∆F elicited antibody responses with demonstrable antibody effector function (Fig. 4, E and F). However, it is not clear how these responses differ from those seen in unvaccinated animals in the context of infection. To compare the magnitude of antibody effector functions in vaccinated and unvaccinated hamsters, plasma was collected from hamsters serially euthanized 1, 4, or 6 dpi (either IN or IP) and analyzed in ADCD and antibody-dependent cellular phagocytosis (ADCP) function assays. Independent of challenge route and vaccination period length, Fc-mediated function assays targeting either N or G showed significantly increased responses in vaccinated hamsters than in unvaccinated hamsters. Activity in both the ADCD (Fig. 8, A and B) and ADCP assays was significantly higher in all vaccinated animals (Fig. 8, C and D). Levels increased from 1 to 6 dpi for both antigens but were more pronounced for N than G; longer vaccination period lengths were associated with an increased response rate in both IN-challenged and IP-challenged animals, supporting a contribution by Fc-mediated effector function to protective efficacy.

Cell-mediated immunity (CMI) may also contribute to NiV vaccine–mediated protection (*43*), and marked elevation of activated CD8^+^ T cells has been reported in survivors of infection (*44*). To characterize T and natural killer (NK) cell populations in VRP-vaccinated hamsters following challenge, spleen samples collected 6 dpi from animals vaccinated at various intervals (3, 7, 14, or 28 days) before challenge were evaluated by flow cytometry (Fig. 8E and fig. S4). CD3^+^, CD4^+^, and CD8^+^ cell levels were highest in hamsters vaccinated 3 days before challenge. We showed that in this shortest vaccine interval group, virus replication is transiently detected after challenge (Fig. 7); a more pronounced immune response is likely due to replication and a priming effect from the recent vaccination. Relative increases were also seen in groups vaccinated at longer intervals, but the differences compared to unvaccinated hamsters decreased as the interval between vaccination and challenge increased, and corresponding virus replication was shown to be lower. NK cell (CD94^+^MHCII^−^) and virus levels were directly associated; NK cell levels were highest in unvaccinated animals and dropped in longer interval vaccination groups shown to better control replication (Fig. 7).

### A single dose of NiV∆F given up to 3 days before challenge is safe and confers complete protection from NiV in an immunocompromised mouse model of disease

**Fig. 9.**
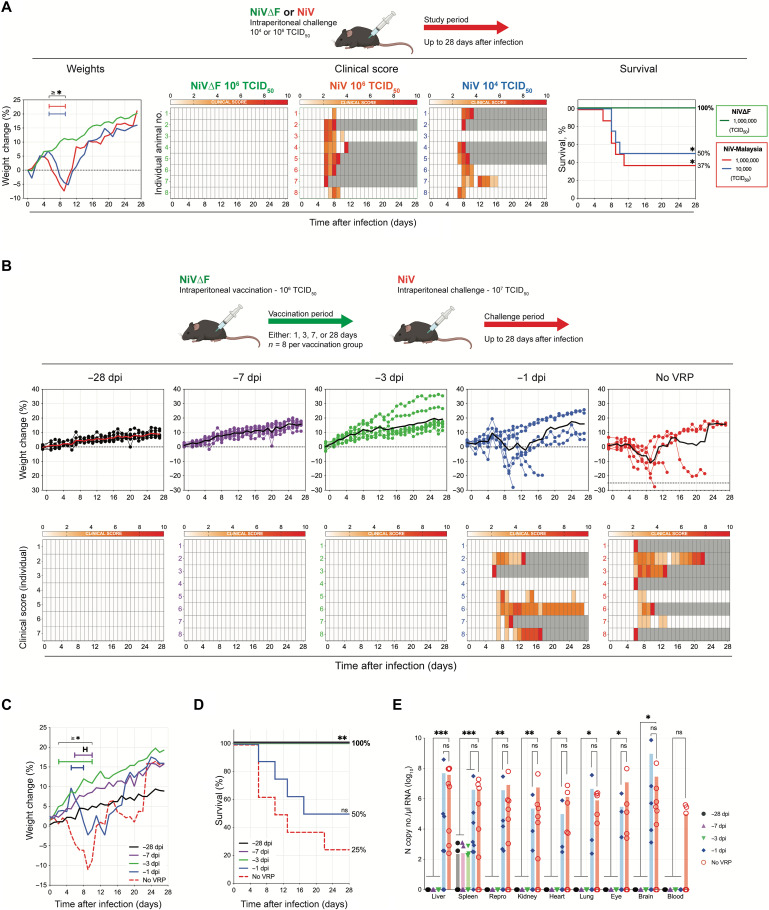
A single dose of NiV∆F provides complete protection from clinical signs and lethality in mouse model of disease up to 3 days before challenge. (**A**) Safety study: Groups of eight Ifnar^−/−^ mice were challenged IP with either NiV∆F (10^6^ TCID_50_, green), or NiV strain Malaysia (10^6^ TCID_50_, red; or 10^4^ TCID_50_, blue), and (**B**) efficacy study: Groups of 8 Ifnar^−/−^ mice were vaccinated IP with 10^6^ TCID_50_ NiV∆F either 28 (black), 7 (purple), 3 (green), or 1 (blue) day before challenge; or mock-vaccinated with DMEM 28 (red) days before challenge. Mice were challenged IP with 10^7^ TCID_50_ of NiV strain Malaysia. Graphs show the percent weight change from baseline (taken −1 dpi; significance calculated by two-way ANOVA: *P ≤ 0.05); individual daily clinical scores (from 0 to 10), with severity depicted by increased intensity of red. Animals scoring ≥ 10 were humanely euthanized; any animals that succumbed to disease before euthanasia were allocated a score of 10. Gray boxes indicate the end of monitoring/scoring due to euthanasia or death. (**C**) Mean percentage weight changes from baseline (taken −1 dpi; significance calculated by two-way ANOVA: *P ≤ 0.05). (**D**) Kaplan-Meier curves show survival in challenged animals; significance calculated by log-rank (Mantel-Cox test): **P ≤ 0.01. (**E**) RT-qPCR detection of NiV vRNA in select tissues. Individual animals from each group are represented. Error bars represent the means and SD. Significance calculated by *t* test: ***P ≤ 0.001; **P ≤ 0.01; *P ≤ 0.05.

We demonstrated lack of clinical disease and the corresponding high safety margin of NiV∆F in suckling mice (Fig. 2A). Immunocompromised Ifnar^**−/−**^ mice are also susceptible to severe and lethal NiV disease. For safety evaluation, mice were inoculated IP with NiV∆F (10^6^ TCID_50_) or NiV (10^6^ or 10^4^ TCID_50_) (each group, *n* = 8; 4 males and 4 females; 37 to 44 days old) and monitored daily (Fig. 9A). Control mice infected with NiV displayed significant weight loss between 5 and 10 dpi. At NiV dose of 10^4^ TCID_50_, seven of eight mice (88%) had clinical signs, and four of eight (50%) succumbed to disease. At the higher dose of 10^6^ TCID_50_, eight of eight mice (100%) had clinical signs, and five of eight (63%) succumbed to disease. In contrast, mice inoculated with 10^6^ TCID_50_ NiV∆F all survived and had no apparent clinical signs throughout the 28-day study period.

To gain further insight into mechanisms of protection and evaluate efficacy in a second model of disease, we next administered NiV∆F using four vaccination periods (28, 7, 3, and 1 day before challenge) in the Ifnar^**−/−**^ mouse model. Groups of eight mice were vaccinated parenterally via the IP route with a single dose of NiV∆F (10^6^ TCID_50_) at indicated time points, or mock-vaccinated (DMEM only at −28 dpi) and subsequently challenged IP with 10^7^ TCID_50_ of NiV (Fig. 9, B to D). All mock-vaccinated mice exhibited weight loss starting at 3 or 4 dpi, all had clinical scores for two or more days, and six of eight (75%) succumbed to infection. While there was delay in percentage weight loss (between 5 and 8 dpi) in mice after a 1-day vaccination period compared to the mock-vaccinated mice, the occurrence of clinical signs, overall lethality, and vRNA levels was comparable. In contrast, mice vaccinated 28, 7, and 3 days before challenge maintained weight, exhibited no clinical signs, and survived (100% of animals) (Fig. 9, C and D). No vRNA was detected in the blood of vaccinated animals nor in any tissues from mice vaccinated 28, 7, or 3 days before challenge except for very low levels in the spleen at study end point (Fig. 9E). Overall, these data confirm high-level efficacy following short- or long-course vaccination and a lack of dependence on early nonspecific IFN-mediated innate responses to confer short-course protection.

## DISCUSSION

Here, we demonstrate high protective efficacy of NiV∆F, a new VRP-based vaccine platform that combines the advantages of a live-attenuated virus platform expressing multiple viral antigens with an inherently high safety profile due to the complete absence of the F gene. The envelopes of NiV∆F virions contain both viral F and G proteins, can enter cells similarly permissive to authentic NiV, and further undergo authentic viral transcription and translation processes. However, any further nascent virion formation is highly inefficient (*45*), and any such particles lack the surface F protein because NiV∆F does not contain the gene for it. These virions could bind to subsequent target cells expressing the ephrin B2 or B3 receptors (*46*–*48*) but would be unable to fuse with and enter these cells. Correspondingly, we demonstrate that the NiV∆F platform is exceptionally safe and highly efficacious. Safety was evaluated in three animal models, and protection against wild-type NiV infection was demonstrated in two. Of the parameters evaluated, NiV∆F VRP meets or exceeds preferred characteristics for intended use, efficacy (both in preventing disease and onset of protection), and dose regimen as detailed in the World Health Organization Target Product Profile for a human NiV vaccine (*49*).

A mucosal vaccine against henipaviruses has not yet been developed. Parenteral vaccination primarily induces a systemic immune response, whereas mucosal delivery can induce both mucosal and systemic immunity (*50*, *51*). Eliciting mucosal immunity is highly advantageous, especially against respiratory pathogens like NiV that enter via mucosal surfaces. We found that both parenteral and intranasal vaccinations with NiV∆F confer high-level protection. The availability of a mucosal vaccine for NiV would greatly advance prevention efforts. Mucosal vaccines have demonstrable utility for other respiratory pathogens, including influenza, SARS-CoV-2, and respiratory syncytial virus (*52*–*55*). In addition to immunogenicity, other benefits of mucosal vaccines include ease of use and noninvasive delivery, which can reduce vaccination hesitancy (*56*, *57*). Furthermore, intranasal delivery device technology is rapidly evolving, presenting novel options for clinical advancement of mucosal vaccine platforms (*50*, *58*, *59*).

After a single dose, NiV∆F elicits broad anti-N, anti-F, and anti-G humoral responses, including IgG and IgA subtypes, and confers protection as little as 3 days following delivery in both hamsters and mice. A recent report on short-course efficacy of a vesicular stomatitis virus–vectored vaccine against NiV suggests that a stronger humoral response may be needed to control infection when vaccine is administered at shorter intervals (*60*). Although IgG antibody responses were first detected 14 days after mucosal NiV∆F vaccination, our data demonstrating increased antibody effector functions early after challenge (1, 4, and 6 dpi) in vaccinated animals also support a role for antibody responses in short-course efficacy. This rapid response is likely mediated by early IgM antibody generation preceding IgG and prompt antibody elicitation after challenge in vaccinees due to priming by recent NiV∆F delivery. Although nonspecific innate responses could also conceivably contribute to short-course protection, the absence of innate immune induction in hamsters and high-level protection in Ifnar^−/−^ mice that lack type I IFN receptor function indicates that innate responses likely play a less prominent role in protection.

Mechanisms of vaccine-mediated protection vary with timing of administration, as longer-course vaccination allows for maturation and development of a potent adaptive response (*61*). Induction of neutralizing antibodies has long been considered a critical determinant of virus vaccine efficacy. However, more recent studies, like those of SARS-CoV-2, have demonstrated effective viral control and protection against tissue pathology without neutralizing antibodies by vaccines that elicit potent T cell responses (*62*) and have highlighted the importance of Fc-dependent effector functions for maximal protection against infection (*41*). In addition, SARS-CoV-2 studies have shown that antibodies capable of ADCP and other Fc-dependent effector functions can serve as predictors of efficacy of convalescent plasma therapy (*63*, *64*). Although neutralizing antibodies were previously thought to be critical for protection against NiV infection and vaccine development efforts to date have focused on their elicitation (*65*–*67*), protection against henipavirus infection requires both a cell-mediated and a humoral immune response (*43*). Vaccination against other paramyxoviruses, such as measles and mumps, elicits both antibodies and CMI (*68*–*70*). While anti-H antibodies serve as the clinical correlate of protection for measles, the correlates of protection for mumps remain unclear. Neutralizing antibody titers are the best indicator of protection, but lack of standardization hinders the use of this measure, and while T cell immunity is detectable, whether it correlates with protection is unknown (*71*, *72*). Here, we find that despite minimal to no detection of neutralizing antibodies after vaccination, NiV∆F was fully protective against NiV, further indicating that nonneutralizing antibodies and cell-mediated responses can play a significant role in vaccine-mediated protection against paramyxoviruses.

NiV G was reported as the immunodominant target for neutralizing antibodies against NiV (*73*), although neutralizing epitopes within F have also been identified (*74*, *75*). Consistent with immunological responses to VRP vaccines we have developed for other viruses, including Lassa (*25*) and Crimean-Congo hemorrhagic fever viruses (*27*, *28*), anti-N antibody responses in NiV∆F-vaccinated animals were higher than those against G or F, which may partly explain the limited induction of neutralizing antibody populations. However, N of various viruses can induce robust T cell responses (*76*–*83*). Moreover, TRIM21 has been shown to use anti-N antibodies to target N for cytosolic degradation and generate cytotoxic T cells against N peptide, linking anti-N antibody responses and T cell function (*84*). Furthermore, other NiV platforms, like the modified vaccinia virus Ankara vaccine expressing recombinant NiV-G, have demonstrated activation of specific CD8 and CD4 T cell responses (*85*). Together, these findings indicate that T cell responses can be generated in response to multiple antigens expressed by the VRP and could correspondingly contribute to protection. While we were able to assess T cell populations, we were not able to investigate other key measures of T cell responses due to the limited availability of reagents and assays for use with hamster samples. T and NK cell population kinetics alone did not explain differences in outcome; future analyses focused on comprehensive evaluation of CMI to VRP vaccination will help elucidate additional contributions to protection.

With the recognized threat to public health posed by NiV, several vaccines have been investigated to date [reviewed in (*86*)], including ones based on adjuvanted protein subunit, recombinant viral vectors, and mRNA (*87*, *88*). Several of these have demonstrated high efficacy in animal models. Like we observed with the VRP, short-course uniform protection (up to 7 days after vaccination) was also demonstrated with a subunit platform based on the Hendra virus attachment glycoprotein ectodomain (*89*), further supporting short-course potential for NiV vaccines. Most vaccine efforts have focused on eliciting neutralizing antibodies, but some have also evaluated and demonstrated CMI. While our studies provide critical data in NiV vaccine advancement efforts, they have limitations, which can hopefully be addressed in future investigation of the VRP. Notably, while the hamster model translates well to the human spectrum of disease, it faces substantial constraints with reagent availability, which restricted full investigation into immunogenicity and correlates of protection. This prevents a detailed comparison to immune responses from other vaccines for better understanding platform-independent correlates of protection. Last, while our findings are incredibly promising, as is the case in all animal model studies, how immunogenicity and protection data will translate to human infection is yet unknown.

VRPs have been developed for a variety of pathogens as vaccines but are also used for many other key applications. Here, we focus on NiV∆F as a highly efficacious vaccine; in addition, we provide the first report on generating a henipavirus VRP system. The VRP platform represents an extremely beneficial tool for the henipavirus field, in particular because it can be used at lower containment levels. In contrast to other systems that isolate single processes, such as pseudotyped viruses or virus-like particles for studying entry (*90*–*92*) or minigenome systems for studying replication (*93*–*96*), VRP is a nonspreading system that recapitulates the full spectrum of viral processes and can be used for many crucial applications, including serology and neutralization assays (*97*–*103*), viral pathogenesis studies (*104*–*106*), and therapeutic screening (*107*–*109*).

Together, we report the development of a highly efficacious mucosal vaccine against NiV and provide a key molecular tool to the field. These studies highlight the need for continued investigations into the spectrum of immunological responses to henipavirus infection and into the relative contributions of neutralizing antibodies, nonneutralizing antibodies, and T cells in vaccine-mediated protection. Future efforts should focus on continued clinical advancement of the NiV∆F vaccine and aim to gain further understanding of the multifactor immune-mediated responses critical for protection against henipavirus infection and disease.

## MATERIALS AND METHODS

### Biosafety and ethics

All work with infectious virus or infected animals was conducted in a biosafety level 4 (BSL-4) laboratory at the Centers for Disease Control and Prevention (CDC) following established BSL-4 standard operating procedures approved by the Institutional Biosafety Committee. All recombinant virus work was approved by the CDC Institutional Biosafety Committee. All animal experiments were approved by the CDC Institutional Animal Care and Use Committee (2956SPEHAMC, 3004SPEMOUC, 3220SPEMULC) and performed in an Association for Assessment and Accreditation of Laboratory Animal Care (AAALAC) International–approved facility.

### Cells and viruses

Vero (CDC Core facility) and BSR-T7/5 [gift from K. Conzelmann, Ludwig-Maximilians-University (*110*)] cells were cultured in DMEM (Thermo Fisher Scientific, 11965084) supplemented with 5% (v/v) fetal calf serum, nonessential amino acids (Thermo Fisher Scientific, 11140050), 1 mM sodium pyruvate (Thermo Fisher Scientific, 11360070), 2 mM l-glutamine (Thermo Fisher Scientific, A2916801), penicillin (100 U/ml), and streptomycin (100 μg/ml) (Thermo Fisher Scientific, 15070063). CHO-K1 cells (Invitrogen, R75807) were cultured in Ham’s F12 medium (Thermo Fisher Scientific, 11765054) similarly supplemented with 10% fetal calf serum, 2 mM l-glutamine, penicillin (100 U/ml), and streptomycin (100 μg/ml). The coding sequences for codon-optimized forms of the viral proteins from NiV strain Malaysia (Genbank, AF212302) were inserted into the genome of CHO-K1 cells and Vero cells using the Flp-In system (Invitrogen, K601001). NiV strain Malaysia (GenBank accession no. AF212302) was originally obtained from a clinical isolate, passaged once on Vero-E6 cells for isolation from clinical sample, and further amplified on Vero cells. NiV titers were calculated as TCID_50_ in Vero cells (*111*). All viral stocks were verified by NGS and confirmed to be mycoplasma-free.

### Generation of NiV VRP

To generate the NiV∆F rescue plasmid, the entire F coding sequence was removed from the full-length NiV rescue plasmid by excision PCR; after agarose gel purification, the amplicon was religated using In Fusion assembly methods (Takara Bioscience, 639650). Correct assembly of the NiV∆F genome was confirmed by NGS of the plasmid. To rescue NiV∆F, we used the established NiV reverse genetics system (*30*), substituting the NiV∆F plasmid instead of full-length genome. Briefly, all required plasmids were transfected into BSR-T7/5 cells, followed by overlaying the transfected cells 2 days later with the F-expressing Vero-Fco cells (P0 stock). Five days after transfection, clarified supernatants were transferred to fresh Vero-Fco cells to generate large viral stocks (P1). All NiV∆F stocks were verified by NGS and confirmed to be mycoplasma-free.

### Electron microscopy

For thin section preparations, Vero cells were infected with NiV strain Malaysia, and Vero-Fco cells were infected with NiV∆F (both at multiplicity of infection 0.01). Cells were harvested 3 dpi, and virus was inactivated by fixation with phosphate-buffered 2.5% glutaraldehyde followed by γ-irradiation (5 × 10^6^ rad from a ^60^Co source). Samples were postfixed in 1% osmium tetroxide, stained en bloc with uranyl acetate, dehydrated in a graded series of alcohols and acetone, and embedded in an Epon substitute/Araldite mixture. Sections were stained with uranyl acetate and Reynolds lead citrate and examined using a Thermo Fisher Scientific/FEI Spirit electron microscope. For negative stain preparations, supernatants from NiV∆F-inoculated Vero-Fco cells were γ-irradiated, banded and concentrated, and resuspended in dH_2_O or 2% buffered paraformaldehyde.

### Hamster studies

For vaccination with NiV∆F, hamsters (male and female; 5- to 7-week-old HsdHan:AURA Syrian hamsters; Envigo, 8903F or 8903M) were inoculated IN with 10^6^ TCID_50_ (100 μl divided bilaterally between the nares using a P200 pipette (Rainin Pipet-Lite XLS LTS). For challenge with NiV, hamsters were inoculated either IN (10^6^ TCID_50_ in 100 μl, divided bilaterally between the nares) or IP (10^4^ TCID_50_ in 500 μl, divided bilaterally). DMEM was used for all mock-vaccinated or mock-challenged animals, administered as above. Hamsters were housed in a climate-controlled laboratory (68° to 79°C and 30 to 70% humidity) with a 12-hour day/night cycle, provided Teklad global 18% protein rodent diet (Envigo) and water ad libitum, and group-housed on corn cob bedding (Bed-o’Cobs ¼″, Anderson Lab Bedding) with cotton nestlets and crinkle paper (Enviro-dri; Shepherd Specialty Papers) in an isolator-caging system (Tecniplast GR900) with a high-efficiency particulate air (HEPA)–filtered inlet and exhaust air supply. Microchip transponders [Bio Medic Data Systems (BMDS), IPTT-300] were placed subcutaneously in the interscapular region for individual identification and to assess body temperature. Insertion sites were closed with GLUture topical tissue adhesive (MWI Veterinary, 034207). Baseline weights were taken 1 day before challenge (−1 dpi), and hamsters were assessed daily after inoculation for weight change, temperature, and clinical signs. Animals were scored according to the following criteria: quiet, dull, responsive (QDR) disposition, hunched back/ruffled coat, hypoactivity, mild neurological signs—2 points each; abnormal breathing (i.e., increased respiratory rate, dyspnea), hypothermia (<34°C), moderate neurological signs—5 points each; paralysis, frank hemorrhage, moribund, weight loss >25% of baseline (measured at −1 dpi), severe neurological signs—10 points each. Neurological signs were classified as: mild—abnormal gait or movement, mild (~0° to 30° from vertical) or sporadic head tilt; moderate—tremors, ataxia, circling, absence seizures, moderate (~30° to 90° from vertical) and persistent head tilt with retained ability to walk, feed, and drink; and severe—limb paralysis, tonic clonal seizure, inability to right, severe (>90° from vertical) head tilt. Hamsters were euthanized with isoflurane vapor when they met euthanasia criteria (score ≥ 10) or at the completion of the study (28 dpi).

### Mouse studies

For NiV∆F safety studies in suckling mice, litters of 2- to 3-day-old CD-1/ICR mice from timed pregnant dams received at embryonic day 15 (E15) to E16 (Charles River, 022CD1) were inoculated IC with 20 to 30 μl of either NiV∆F or NiV strain Malaysia at indicated doses (*n* = 9 to 13; dose range: 1 to 10^6^ TCID_50_). For NiV∆F safety studies in Ifnar^−/−^ mice, male and female 5- to 6-week-old B6.129S2-Ifnar1tm1Agt/Mmjax mice (MMRRC/The Jackson Laboratory, 032045-JAX-HOM-F and 032045-JAX-HOM-M) were inoculated IP (200 μl in total) with 10^6^ TCID_50_ of either NiV∆F or NiV strain Malaysia. For vaccine efficacy studies, Ifnar^−/−^ mice (male and female; 5 to 10 weeks old at time of challenge) were vaccinated with NiV∆F IP as above and challenged IP with 200 μl of either NiV∆F or NiV strain Malaysia (10^7^ TCID_50_). As described for hamster studies, mice were group-housed on corn cob bedding with cotton nestlets and crinkle paper in an isolator-caging system (Tecniplast GM500 or GR900) with a HEPA-filtered inlet and exhaust air supply, in a climate-controlled laboratory (68° to 79°C and 30 to 70% humidity) with a 12-hour day/night cycle, and provided sterilized commercially available rodent feed (Laboratory Autoclavable Rodent Diet 5010, LabDiet) and sterile water ad libitum. Baseline weights for the challenge period were obtained −1 dpi, and mice were monitored daily for 28 dpi. Clinical signs in mice were scored on the basis of 18 parameters: 2 points each for QDR disposition, hunched back and/or ruffled coat, hypoactivity, mild neurological, or mild respiratory signs; 3 points each for dehydration or abnormal huddling; 5 points each for moderate neurological signs (e.g., ataxia/circling/tremors/paresis), moderate respiratory signs (e.g., moderate dyspnea), or anemia; 7 points for weight loss of >20%; and 10 points each for inability to bear weight, severe neurological signs (e.g., paralysis), severe respiratory signs, frank hemorrhage, moribund state, or weight loss of >25%. Animals were humanely euthanized with isoflurane vapors followed by cervical dislocation when end-point criteria were reached (clinical score ≥ 10) or at study completion (28 dpi).

### RT-qPCR assays for viral quantification and relative immune transcript level calculations

vRNA was extracted from homogenized tissue samples (liver, spleen, gonad, kidney, heart, lung, eye, and brain), whole blood, and mucosal swabs samples (oral and rectal) using the MagMAX-96 Total RNA Isolation Kit (Thermo Fisher Scientific, AM1830) on a 96-well ABI MagMAX extraction platform. Genomic DNA was removed using BaseLine Zero DNase (Biosearch Technologies, DB0715K), and RT-qPCR was performed using SuperScript III Platinum One-Step RT-qPCR Kit (Thermo Fisher Scientific, 11732088) with strain-specific primers and probe targeting NiV N gene (data file S1), with levels normalized to 18*S* RNA values using a commercial endogenous control assay (Thermo Fisher Scientific, 4310893E). Reactions to quantify NiV N vRNA levels were performed in technical duplicate. Genome copy numbers were determined using standards prepared from in vitro–transcribed N RNA and performed in technical triplicate. Assays to determine differentially expressed immune genes (data file S1) in hamsters were designed (all Integrated DNA Technologies) and run with SuperScript III Platinum One-Step RT-qPCR Kit according to the manufacturer’s instructions. Cytokine expression levels in lung tissue RNA were calculated using ∆∆Ct (with B2M RNA levels housekeeping control) relative to mock-inoculated (DMEM only) control hamsters sacrificed at similar time points after inoculation (reactions performed in technical triplicate).

### Protein expression and purification

The sequence of NiV N (GenBank accession no. AK50540.1) was optimized for bacterial expression and cloned into pET28a with N terminal 8× His tags and 3C cleavage site by Twist Bioscience (*112*). The construct was transformed into *Escherichia coli* BL21 (DE3) strain (Thermo Fisher Scientific, EC0114), and bacterial culture was grown in Luria broth with kanamycin. The culture was induced with 1 mM isopropyl β-d-1-thiogalactopyranoside when the optical density was 0.4 to 0.6. Following induction, the culture was transferred to 16°C for overnight incubation. Cells were harvested with centrifugation, resuspended in lysis buffer [500 mM NaCI, 20 mM tris-CI (pH 7), 0.1% Triton X-100 (v/v), 5% glycerol (v/v), 1 M urea, and 1× protease inhibitor cocktail], and sonicated. Cleared bacterial lysates were filtered through a 0.2-μm polyethersulfone membrane and loaded onto HisTrap Excel columns (Cytiva, 17371205) for immobilized metal affinity chromatography. Fractions containing NiV N protein were pooled, concentrated, and dialyzed against phosphate-buffered saline (PBS) using Amicon centrifugal filters (Thermo Fisher Scientific, UFC905096). The sequences of the stabilized pre-fusion NiV F glycoprotein (GenBank accession no. AAK50544.1) with a C-terminal GCN4 domain-3C cleavage site-8× His tag-twin Strep tag and the soluble head domain of NiV G (residues 171 to 602; GenBank accession no. AAK50545.1) with C terminal 3C cleavage site-8× His tag-twin Strep tag were cloned into pEEV plasmid by Twist Bioscience (*113*, *114*). The proteins were expressed in Expi293F cells (Thermo Fisher Scientific, A14527) growing in Expi293 expression medium via transient transfection using FectoPro transfection reagent according to the manufacturer’s recommendations (Polypus, 101000007). Briefly, cells were transfected with 0.8 μg plasmid/ml of culture using 1.5 μl of transfection reagent/μg of the plasmid. Four to 6 days after transfection, culture supernatants were clarified, filtered, and loaded onto the HisTrap Excel column. NiV F and G proteins were further purified via size exclusion chromatography (Superdex 200 increase 16/600 GL, Cytiva, 28989335). Following purification, proteins were quantified and stored at −80°C.

### Enzyme-linked immunosorbent assay

Maxisorp plates (Thermo Fisher Scientific, 442404) were coated overnight at 4°C with recombinant NiV Malaysia N or GP diluted in sterile-filtered coating buffer [Na_2_CO_3_ (Sigma-Aldrich, 222321), 1.5 g; NaHCO_3_ (Sigma-Aldrich, S6014), 2.93 g; and quantum satis 1 L diH_2_O (pH 9.6)] Coated plates were washed five times using Tris-buffered saline-Tween-20 (TBS-T, Thermo Fisher Scientific, 28360) and a Biotek automated plate washer. Wells were blocked for 1 hour at 37°C with blocking buffer [5% milk (Cell Signaling Technology, 9999S) in wash buffer (TBS-T)]. Serially diluted plasma in blocking buffer was added to the blocked plates and incubated for 1 hour at 37°C. Plates were washed five times before secondary antibody was added [horseradish peroxidase–conjugated goat monoclonal anti-hamster IgG (Invitrogen, PA1-29626)] diluted in blocking buffer and incubated for 1 hour at 37°C. After washing, TMB-Ultra solution (Thermo Fisher Scientific, 34029) was added, and plates were incubated at room temperature for 6 min. Reactions were stopped with Stop solution (Invitrogen, SS04), and 450-nm absorbance was read on a Biotek plate reader. Each reaction was performed in technical triplicate from individual animal samples.

### Virus neutralization

To determine levels of NiV-specific neutralizing antibodies, twofold dilutions of hamster plasma (final plasma dilution range 1:4 to 1:2560) were incubated with 200 TCID_50_ of NiV strain Malaysia for 1 hour and then transferred onto Vero cells for 7 days at 37°C. NiV-neutralizing titers were recorded as the lowest reciprocal dilution in which all four wells were protected from cytopathic effects. Each reaction was performed in technical quadruplicate.

### Antibody-dependent complement deposition

Recombinant NiV strain Malaysia N and G proteins were biotinylated (EZ-Link Sulfo-NHS-LC-Biotinylation Kit, Thermo Fisher Scientific, 21435) and coupled to 1.0-μm red fluorescent neutravidin microspheres (FluoSpheres NeutrAvidin-Labeled Microspheres, Thermo Fisher Scientific, F8775). Excess antigen was washed away with PBS containing 5% bovine serum albumin (BSA). Antigen-coated beads were incubated with hamster plasma (2 hours at 37°C), and unbound antibodies were washed away with PBS. Guinea pig complement (Cedarlane, CL4051) diluted in gelatin veronal buffer (CompTech, B102) was added and incubated 15 min at 37°C. Immune complexes were washed with 15 mM EDTA in PBS and incubated 15 min at room temperature with fluorescein isothiocyanate (FITC)–conjugated goat IgG fraction to guinea pig complement C3 (MP Biomedicals, 0855385). Unbound antibody was washed away with PBS, and immune complexes were analyzed on a Guava cytometer. Fold ADCD activation was calculated on the basis of naïve hamster plasma. Each reaction was performed in technical duplicate.

### Antibody-dependent cellular phagocytosis

Immune complexes were formed as described for ADCD, but biotinylated antigen was coupled to 1.0 μm green fluorescent neutravidin microspheres (Thermo Fisher Scientific, F8776). Excess antigen was washed away with PBS containing 5% BSA. Antigen-coated beads were incubated with hamster plasma (2 hours at 37°C), and unbound antibodies were washed away with PBS. Immune complexes were incubated overnight at 37°C with 1 × 10^4^ THP1 cells per well. The next day, cells were washed and analyzed on a Guava cytometer. Phagocytic score was calculated by multiplying the percentage of bead-positive cells with the overall median fluorescence intensity and dividing that number by 10,000. Each reaction was performed in technical duplicate.

### Flow cytometry

Flow cytometric analysis was performed on spleens collected from hamsters at necropsy and processed into a single-cell suspension by homogenization through a 70-μm mesh filter. Red blood cells (RBCs) were lysed using RBC lysis buffer (Sigma-Aldrich, 11814389001), and splenocytes were resuspended in freezing media [10% dimethyl sulfoxide + heat-inactivated fetal bovine serum (FBS)] and stored in liquid nitrogen until further analysis. After thawing in the presence of Benzonase (0.5 U/ml; EMDmillipore, E1014-5KU) in RPMI 1640 (Thermo Fisher Scientific, 11875085) supplemented with 10% heat-inactivated FBS, penicillin (100 U/ml), and streptomycin (100 μg/ml), splenocytes were rested overnight at 37°C and 5% CO_2_. The following day, splenocytes were incubated with NiV peptides spanning the N (Q9IK92) and G (Q9IH62) genes (AbClonal) in the presence of GolgiPlug (BD Biosciences) for 6 hours. Splenocytes were washed and incubated with blocking buffer [2% mouse serum and 2% rat serum (both from Jackson Immunoresearch) diluted in PBS containing 1% heat-inactivated FBS] and subsequently incubated with surface stains [MHCII-BV711, clone 14-4-4S (BD Biosciences, 745465); CD4-allophycocyanin (APC)-Cy7, clone GK1.5 (BL 100414); CD8-phycoerythrin (PE), clone 341 (Thermo Fisher Scientific, 12-0080-82, eBioscience); CD94-PE, clone 18d3 (BL 105508)] for 30 min on ice. Cells were washed and resuspended in Cytofix/Cytoperm (BD Biosciences) for 20 min at room temperature, followed by a permeabilization step and intracellular staining [CD3-FITC, clone CD3-12 (Abcam, ab34722)]. Cell viability was determined using Tonbo Ghost Dye Violet 510. After additional washes, cells were resuspended in fluorescence-activated cell sorting buffer (PBS with 1% heat-inactivated FBS) and analyzed on a Stratedigm S1000EXi. Data analysis was performed using FlowJo software (version 10.8.2), and gating strategy is shown in fig. S4.

### Histopathology and ISH

Representative sections of the lung, heart, liver, spleen, kidney, brain, eye, trachea, esophagus, adrenal gland, pancreas, gall bladder, reproductive tissues, stomach, small intestine, and colon, as available, were evaluated by histopathology. Necropsy tissues were fixed in 10% neutral-buffered formalin, γ-irradiated (5 × 10^6^ rad), and processed for routine paraffin histology. Sections were cut at 4 μm, mounted on glass slides, and stained with hematoxylin and eosin for histopathologic evaluation. Unstained tissue sections processed in the same way were used for ISH using RNAscope probes developed to target the nucleocapsid (N) gene (Advanced Cell Diagnostics, 1193901-C1) according to the RNAscope 2.5 Assay protocol (ACD document nos. 322452 and 322360) to localize vRNA and evaluate for virus spread in lung tissues. Paraffin-embedded NiV-infected cells were used as positive controls.

### Graphing and statistical analyses

All graphs were created in GraphPad Prism (v9.4.1). Survival statistics were calculated using the log rank (Mantel-Cox) test. Relative weight loss statistics were calculated using two-way analysis of variance (ANOVA) (mixed-model with Geisser-Greenhouse correction and Dunnett’s multiple comparisons test). RT-qPCR, viral titer, and antibody assay statistics were calculated using multiple *t* tests (Mann-Whitney test, unpaired, nonparametric, and Holm-Šídák method of multiple comparisons). Study schematics were created with BioRender.com.
