# Supplementary material for: Single-dose mucosal replicon-particle vaccine protects against lethal Nipah virus infection up to 3 days after vaccination
Source: Sci Adv. 2023 Aug 4;9(31):eadh4057. doi: 10.1126/sciadv.adh4057 (PMC10403222; doi:10.1126/sciadv.adh4057)
Supplement: Supplementary file 1 — Figs. S1 to S4 [file sciadv.adh4057_sm.pdf]

Science Advances
AAAS

Supplementary Materials for

Single-dose mucosal replicon-particle vaccine protects against lethal Nipah virus infection up to 3 days after vaccination

Stephen R. Welch *et al.*

Corresponding author: Christina F. Spiropoulou, ccs8@cdc.gov

Sci. Adv. **9**, eadh4057 (2023)
DOI: 10.1126/sciadv.adh4057

The PDF file includes:

Figs. S1 to S4

Other Supplementary Material for this manuscript includes the following:

Data file S1

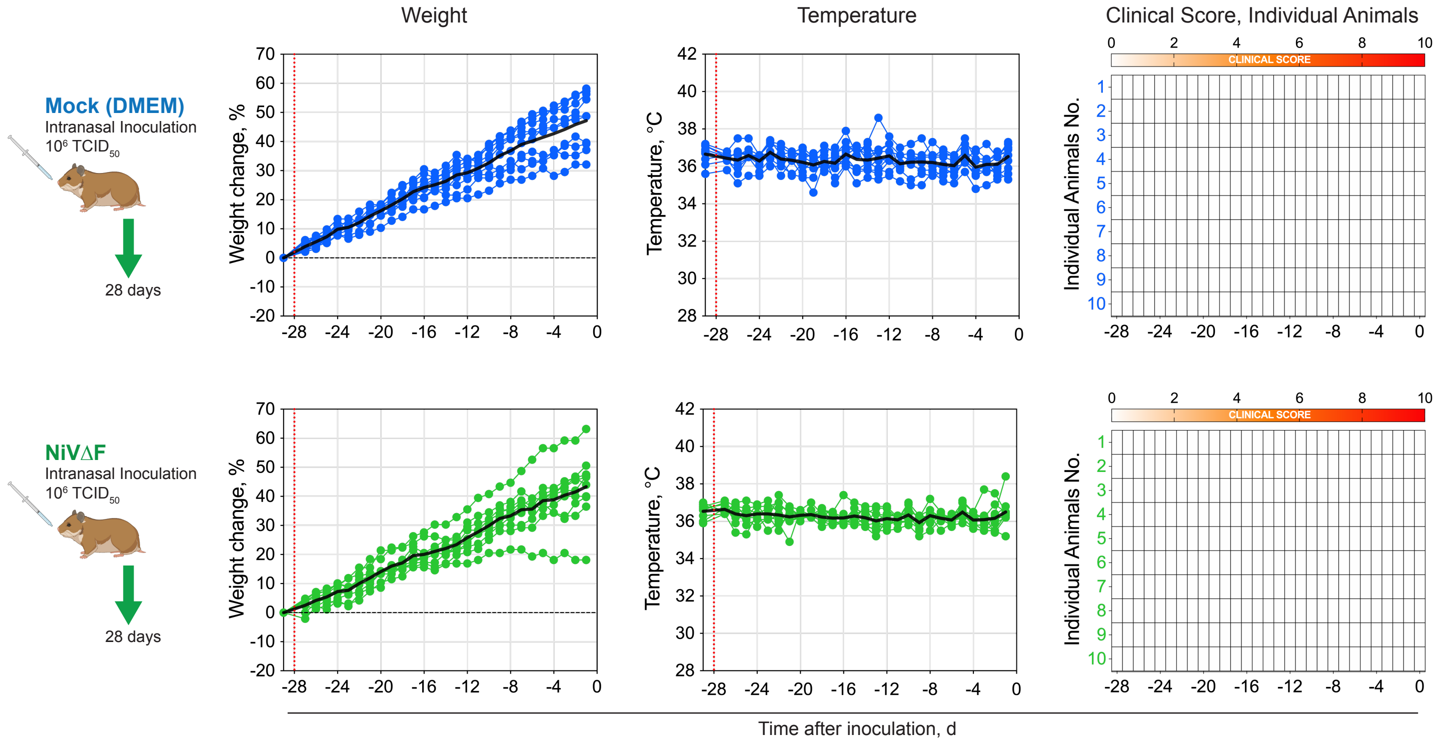

Figure S1: Safety of IN NiVΔF vaccination in the Syrian hamster model of NiV disease.
Groups of Syrian hamsters (n = 10; 5 males, 5 females; all 5 – 7 weeks old) were inoculated IN with either 10^6 $TCID_{50}$ of NiVΔF (n = 10, green circles) or mock-vaccinated with DMEM (n = 10, blue circles) and monitored daily for 28 days. Graphs indicate percentage weight change from baseline (taken −1 dpi), body temperature, and clinical signs (scored from 0 to 10) with severity depicted by increased intensity of red. Individual animals are represented, with the daily mean represented as a solid line.

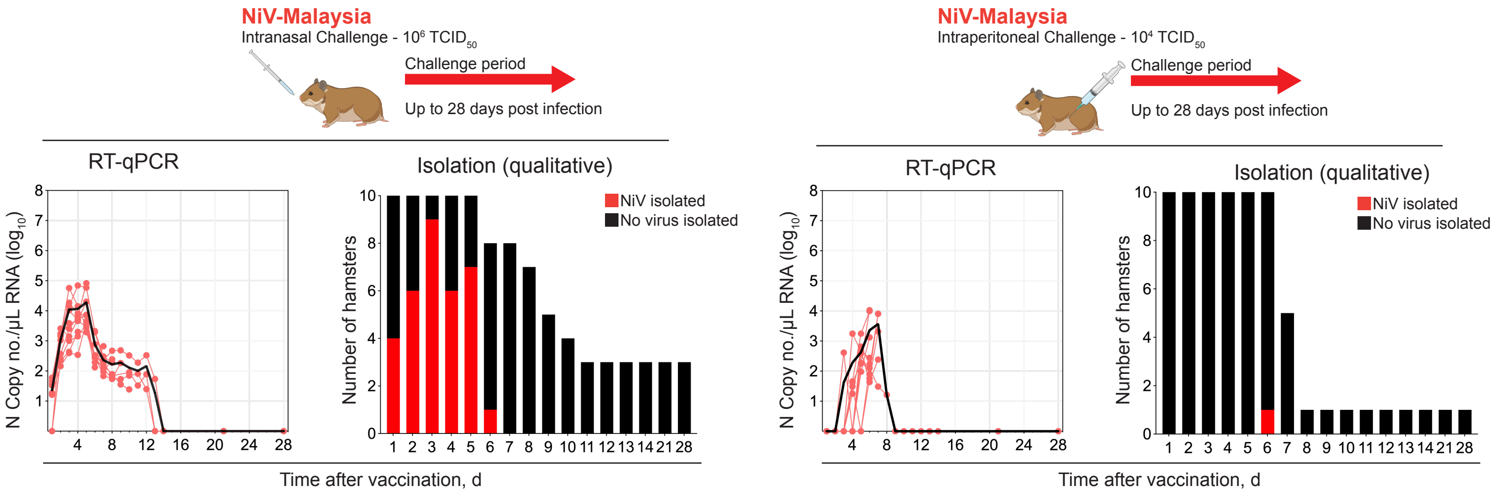

Figure S2: Mucosal shedding in hamsters infected either intranasally or intraperitoneally with NiV. Groups of Syrian hamsters (n = 10; 5 males, 5 females; all 5 – 7 weeks old) were challenged either IN (10^6 $TCID_{50}$) or IP (10^4 $TCID_{50}$) with wild-type NiV. Oral swabs were taken daily 1 – 14 dpi and then again at both 21 and 28 dpi, the latter being study completion. For each swab sample, levels of NiV RNA were quantified by RT-qPCR, and qualitative presence of infectious virus was determined by isolation in cell culture. In both IN- and IP-inoculated animals, NiV RNA levels peaked 6 dpi. NiV was isolated from only 1 of 10 IP-inoculated animals (at 6 dpi), whereas virus was isolated from all 10 IN-inoculated hamsters at least once (between 3 – 5 dpi). Individual animals are represented as red circles, with a solid black line indicating the daily mean. For qualification, results were either scored positive (red) or negative (black) for detection of infectious virus.

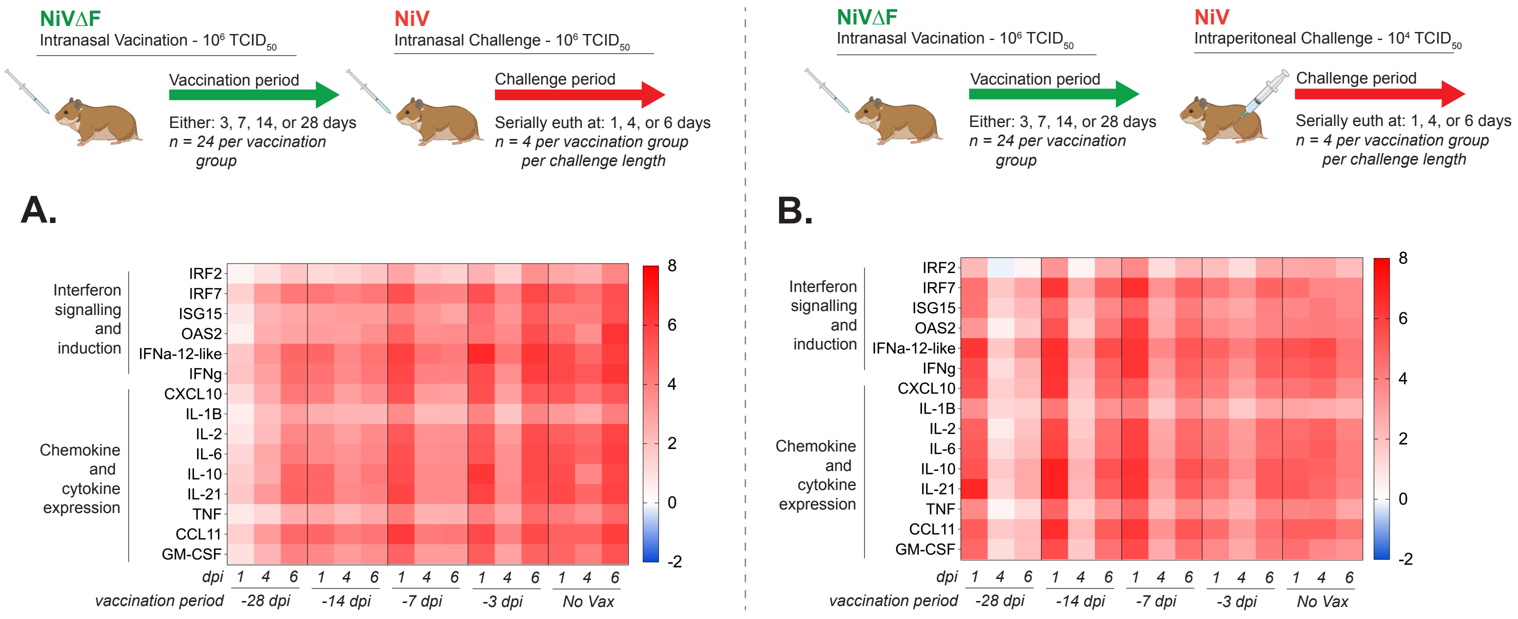

Figure S3: Early Innate immune responses after challenge in lungs of vaccinated hamsters.
Groups of hamsters were vaccinated IN once 28, 14, 7, or 3 days prior to challenge, or mock-vaccinated with DMEM 28 days prior to challenge (n = 120; for each vaccination group, n= 24; 12 males and 12 females; all age matched). Animals were challenged either IN with 10^6 $TCID_{50}$ (n = 60) or IP with 10^4 $TCID_{50}$ (n = 60) with NiV strain Malaysia. At 1, 4, and 6 dpi, select animals were euthanized for analysis (n = 40 per dpi; n = 4 per vaccination period per vaccination route per day post challenge; 2 males and 2 females per group). Heat-maps represent log2 fold-changes in transcript levels (compared to mock-treated hamsters at each day) of mRNA associated with genes from either interferon induction and signaling, or cytokine and chemokine signaling pathways detected in lung RNA. Intensity of color corresponds to magnitude change over baseline.

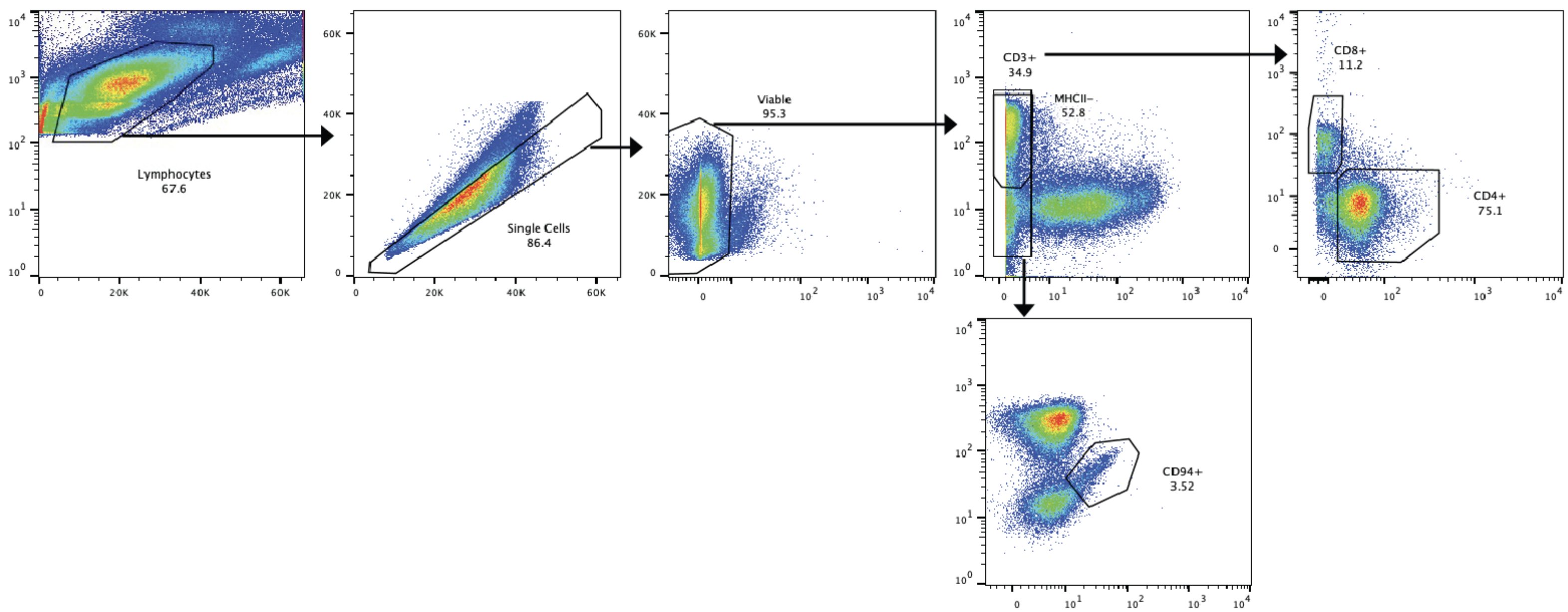

Figure S4: Gating strategy for lymphocyte panels.
Single cell suspensions of splenocytes were initially gated by SSC-A and FSC-A followed by FSC-A and FSC-H to identify single cells. Viable cells were identified by the Live/Dead negative gate. CD3+ T cells were gated by CD3+MHCII− from viable single cells and then subsequently gated by CD4 or CD8. NK cells were gated by CD94+MHCII− from viable single cells. Normal healthy hamsters were used to illustrate the gating strategy.
